# Quantification of upper body movements during gait in older adults and in those with Parkinson’s disease: impact of acceleration realignment methodologies

**DOI:** 10.1016/j.gaitpost.2016.11.047

**Published:** 2017-02

**Authors:** Christopher Buckley, Brook Galna, Lynn Rochester, Claudia Mazzà

**Affiliations:** a MRC-Arthritis Research UK Centre for Integrated Research into Musculoskeletal Ageing (CIMA), Pam Liversidge Building, University of Sheffield, Sheffield S1 3JD, UK; b Department of Mechanical Engineering, University of Sheffield, Sir Frederick Mappin Building, Sheffield S1 3JD, UK; c Institute of Neuroscience/Newcastle University Institute for Ageing, Newcastle University, Clinical Ageing Research Unit, Campus for Ageing and Vitality, Newcastle upon Tyne NE4 5PL, UK; d INSIGNEO Institute for in Silico Medicine, University of Sheffield, Pam Liversidge Building, Sheffield S1 3JD, UK

**Keywords:** Parkinson's disease, Harmonic ratio, Jerk, Inertial sensors, Attenuation

## Abstract

The upper body accelerations of people with Parkinson’s disease (PD) measured by inertial measurement units (IMUs) may contribute towards diagnostic algorithms and help track disease progression. Before extracting variables related to upper body motion, acceleration signals require realignment to a global reference; however, the impact of these techniques on the resulting upper body variables is unclear. Therefore, the aim of this investigation was to examine the impact of four different realignment methods designed to correct acceleration signals on a range of upper body variables in older adults and in patients with PD. Two minutes of continuous gait were measured in 54 community-dwelling older adults (71.1 ± 6.7 years) and 60 people with PD (age: 68.5 ± 9.1 years). Three IMUs placed on the 5th lumbar vertebra, 7th cervical vertebra and the back of the head recorded the acceleration of the upper body. A selection of upper body variables sensitive to impaired upper body control in PD and four acceleration realignment methods were compared. A mixed-model ANOVA showed that the choice of realignment method significantly affected the values of upper body variables as well as their ability to discriminate between the PD and control group. Our findings indicate researchers and clinicians should be cautious when comparing upper body variables extracted from IMUs using different realignment methods, and consideration of realignment technique will be important when identifying the most sensitive markers of disease presence and progression. Therefore, it’s strongly recommend that researchers consider and report their realignment methods when assessing upper body variables during gait.

## Highlights

• Upper body acceleration gait variables can be sensitive to early onset Parkinson’s disease.
• Four upper body acceleration realignment methods were compared during gait.
• Different realignment methods can alter upper body gait variables.
• The realignment method used can determine if a variable is sensitive to discriminate controls from Parkinson’s disease.

## 1 Introduction

Neurodegenerative diseases such as Parkinson’s disease (PD) reduce an individual’s ability to walk safely and efficiently. Consequently, gait quantification is potentially a powerful tool to identify incipient pathology, contribute towards diagnostic algorithms, and quantify disease progression [1], [2]. An emerging perspective is that the movement of the upper body during gait plays a critical role in locomotion and may be impaired for people with a range of pathologies [3], [4], [5]. For example, people with Parkinson’s disease (PD) have symptoms such as increased axial rigidity, flexed posture and asymmetrical arm swing [2], which may affect upper body control [6], [7], [8]. As a result, upper body variables are proposed as potential markers of disease progression and may help define disease phenotypes [9].

Recently, the development of wearable devices such as inertial measurement units (IMUs) has permitted their use for routine gait analysis as they record a range of valid gait and postural control measurements and overcome many limitations of traditionally used gait analysis systems [10], [11]. However when using IMUs, caution is required for upper body analysis since their outputs are relative to their own local reference frames [12]. The likelihood of varying mechanics of the upper body segment between participants, such as a possible increased flexed trunk for people with PD [2], and varying sensor locations upon the upper body does not always allow for uniform alignment between separate sensors’ local reference frames. The absence of uniform alignment therefore provides potential for directional crosstalk errors to occur when comparing different participants, groups and sensor locations [13], [14].

To overcome potential crosstalk problems, different methods have been proposed to align IMU local reference frames to a global reference [14], [15], [16]. This realignment might impact gait variables and their ability to discriminate between patient groups, as shown by a previous study focusing on the pelvis movements [13]. Other upper body variables and segments, not previously investigated, might be similarly affected. Currently, a variety of realignment methods being used in the literature but their influence on the wide selection of upper body acceleration-based variables is still unknown. It can be hypothesised that differences in realignment methods will influence upper body variables for both healthy elderly and people with PD, and that the resultant changes in the upper body variables might differ in the two groups. Therefore, if upper body variables are to be used to quantify gait impairments for people with PD, an understanding of the impact of pre-processing techniques is essential, particularly if results are to be compared across studies [13]. This paper aimed to describe how different methods designed to realign IMUs to a comparable global reference alter acceleration derived upper body variables known to be sensitive to impaired upper body control. To exemplify the potential impact of these alterations, we also investigated whether the choice of realignment method impacts the ability of upper body variables to discriminate PD from age-matched controls.

## 2 Materials and methods

### 2.1 Subjects

Participants were recruited into ICICLE-GAIT within 4 months of diagnosis. This is a collaborative study with ICICLE-PD, an incident cohort study (Incidence of Cognitive Impairment in Cohorts with Longitudinal Evaluation – Parkinson’s disease). ICICLE-GAIT recruited a subset of the cohort. Participants were excluded if they had any neurological (other than PD), orthopaedic, or cardiothoracic conditions that may have markedly affected their walking or safety during the testing sessions. The PD participants had to be diagnosed with idiopathic PD according to the UK Parkinson’s Disease Brain Bank criteria and were excluded if they presented significant memory impairment (Mini Mental State Exam (MMSE) ≤24 [17]), dementia with Lewy bodies, drug induced parkinsonism, “vascular” parkinsonism, progressive supranuclear palsy, multiple system atrophy, corticobasal degeneration, or poor command of English. None of the participants demonstrated severe tremor or dyskinesia. This study was conducted according to the Declaration of Helsinki and had ethical approval from the Newcastle and North Tyneside research ethics committee. All participants signed an informed consent form.

### 2.2 Measurement protocol

Fifty-four community dwelling older adults and sixty people with early onset PD walked at their preferred pace for two minutes around a 25 m circuit containing 7 m long Gaitrite pressure activated electronic walkway (Platinum model Gaitrite, software version 4.5, CIR systems, United States of America) (see Table 1 for participant sand spatiotemporal information) [18]. Upper body accelerations were measured using three IMUs (128 Hz, Opal™, APDM Inc, Portland, OR, USA) located at 5th lumbar vertebra to represent movements at the pelvis level (P), the 7th cervical vertebra to represent movements at the shoulder level (S) and upon the back of the head (H). The sensors were placed so that the X axis pointed downwards representing the vertical direction (V), the Y axis pointed to the left representing the medio-lateral direction (ML) and the Z axis pointed backwards representing the anterior-posterior direction (AP). The Gaitrite and the IMUs were synchronised (±1 sample) using a custom-made cable and the data was collected using the same A/D converter.

### 2.3 Data analysis

Four methods to realign upper body IMU data during gait were compared. All realignment methods have been implemented following the descriptions from the papers where they were proposed and are described here briefly:

**Method 1 (M1):** Raw data was used to represent studies whereby no realignment correction was applied [3], [8]. Variables calculated based upon the uncorrected data were also shown to act as reference to see how the following methods impacted the original signal and the subsequently calculated variables.

**Method 2 (M2):** M2 performs a dynamic tilt correction of the acceleration signal towards the vertical gravitational acceleration axis [14]. For gait data, the method relies upon the assumption that the acceleration in the anterior-posterior (AP) direction are constant (i.e. the AP axis is aligned with the vector representing the subject’s mean velocity of progression, and moving consistently with it).

**Method 3 (M3):** Proposed as a progression from M2, M3 attempts to improve it through removing low-frequency movements not associated to gait such as “head nodding’. This is obtained by continuously aligning measured accelerations with the global coordinate system using pitch and roll corrections about a floating unit vector. These corrections are quantified by identifying the low frequency movements in the original signal using a low-pass filter scaled to one quarter of the step frequency. [15].

**Method 4 (M4):** M4 differs as it uses the IMU’s quaternion output to provide a global reference frame [16], [19]. The method requires a uniform magnetic field throughout the area of testing and a calibration trial to be performed to establish the laboratory’s global reference [19]. Both requirements were checked via assessing the consistency of the quaternion output of all IMU’s throughout the testing area of the laboratory and by placing an external sensor on the floor to create the global reference. The sensor acting as the global reference was placed with its vertical component aligned to the earth’s vertical axis, the (AP) direction aligned to the direction of the participant’s walking direction and the medio-lateral (ML) axis was defined according to a right-handed reference frame. The participant’s gait data was realigned to the quaternion based reference on a single sample basis.

Following realignment, the acceleration data was segmented based upon the foot-contact values obtained from the Gaitrite. The mean value was removed to allow comparison of all signals. A low-pass 4th order Butterworth filter with a cut-off frequency of 20 Hz was applied. Data for each stride was normalised to 100 data points using linear interpolation. Each upper body variable was calculated on a single stride basis. All signals were processed using MATLAB (version 8.4.0, R2014b).

### 2.4 Upper body variables

Upper body variables obtained from acceleration signals that have previously been reported to be sensitive to impaired upper body control and promoted as a discriminative variable for people with PD by those who have implemented each realignment method were selected [9].

**Acceleration Root Mean Square (RMS):** Acceleration RMS was calculated using the root mean square (RMS) of the accelerations, measured by each sensor for each stride and all directions [20].

**Jerk RMS (jerk):** The first time derivate, i.e. the jerk, of each component of the acceleration signal was calculated and its RMS was then computed [21]:

(1) $$jerk=RMS\left(\dot{a}\right)$$

**Harmonic ratio (HR):** The harmonic ratio describes the step-to-step (a)symmetry within a stride which has been found to be reduced as a result of PD [5], [7], [8]. The HR was calculated via discrete Fourier transform for each of the acceleration components measured at the three levels [8]. The fundamental frequency was set equal to the stride frequency.

For the AP and V components, the HR was defined as:

(2) $${HR}_{AP,V}=\frac{\Sigma \text{Amplitudes of even harmonics}}{\Sigma \text{Amplitudes of odd harmonics}}$$

For the ML component the HR was defined as:

(3) $${HR}_{ML}=\frac{\Sigma \;\text{Amplitudes of odd harmonics}}{\Sigma \;\text{Amplitudes of even harmonics}}$$

**Jerk ratio (JR):** The jerk ratio is calculated by making log ratios from the derivatives of the AP, ML and V signals to obtain normally distributed and dimensionless gait parameters [22]:

(4) $$JR_{AP}=10log_{10}\left(\frac{jerk_{AP}}{jerk_{V}}\right)$$

(5) $${JR}_{ML}=10log_{10}\left(\frac{jerk_{ML}}{jerk_{V}}\right)$$

**Coefficients of attenuation (CoA)**: these parameters describe the ability to attenuate accelerations from inferior to superior locations through the upper body [16].

The CoAs were computed using the RMS values of the head (*RMS_H_*), shoulder (*RMS_s_*) and pelvis (*RMS_P_*) as follows [16]:

(6) $${CoA}_{PH}=\left(1-\frac{{RMS}_{H}}{{RMS}_{P}}\right)\times 100$$

(7) $${CoA}_{PS}=\left(1-\frac{{RMS}_{S}}{{RMS}_{P}}\right)\times 100$$

(8) $${CoA}_{SH}=\left(1-\frac{{RMS}_{H}}{{RMS}_{S}}\right)\times 100$$

$CoA_{PH}$ represents the attenuation from the pelvis to the head, $CoA_{PS}$ represents the attenuation from the pelvis to the shoulder and $CoA_{SH}$ represents the attenuation from the shoulder to the head.

### 2.5 Statistical analysis

A mixed-design ANOVA was used to determine if the realignment method affected each characteristic regardless of group (method main effect) and whether the group differences were impacted by the use of the different methods (interaction effect). The within repeated measures factor was defined by the realignment methods. If the residuals from the ANOVA were not normally distributed for each variable, a transformation was performed on the participants’ values to ensure normality. To further investigate the discriminant ability of the investigated variables, and to reduce the chance of occurring a type-1 error, paired samples *t*-tests were only performed for the upper body variables where an interaction occurred. This analysis tested if the two groups differed significantly independent of realignment method. The *p* value (significance set at 0.05) and effect size of each comparison was calculated.

## 3 Results

**Fig. 1:**
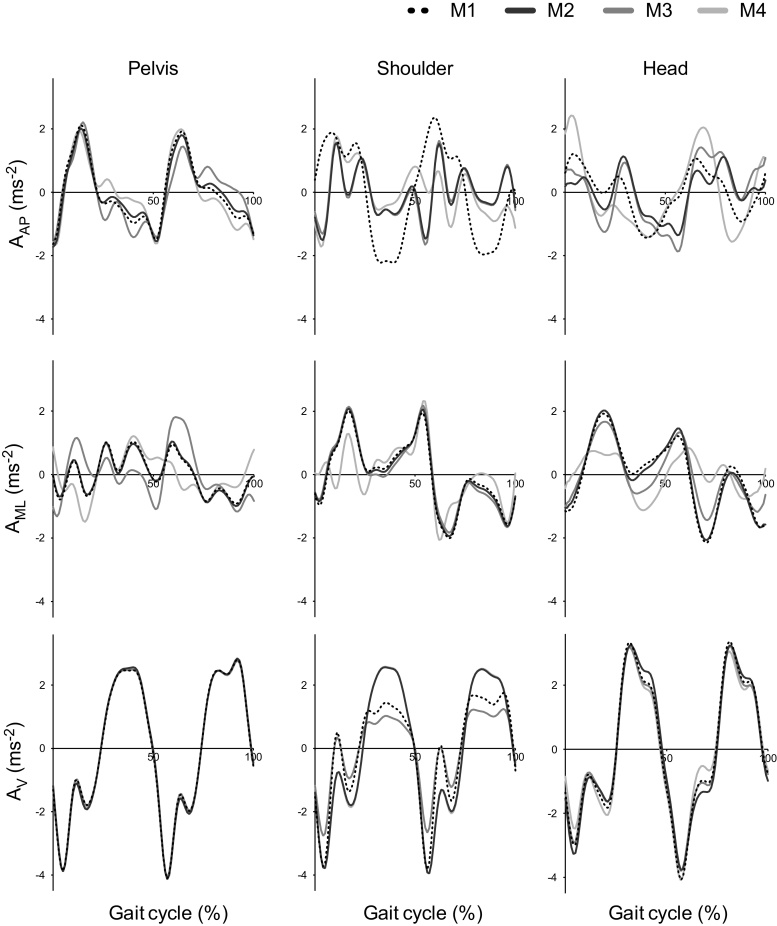
An indication of the method effect upon the acceleration signals measured by the three sensors for one control participant’s stride in the anterior-posterior (A_AP_), medio-lateral (A_ML_) and vertical (A_V_) directions.

Fig. 1 provides a visual example of the impact of each realignment method upon the acceleration signal for all levels and directions analysed during an example stride. Vertical signals varied the least between the realignment methods and the realignment method had most impact for acceleration signals measured at the shoulder level.

There was a significant method main effect for all upper body variables, meaning that each upper body variable differed significantly between the four realignment methods irrespective of group.

Significant interactions were seen for 12 of the 42 upper body variables. All variables are shown for both groups and the four realignment methods (Table 2). Post-hoc analysis revealed that for select upper body variables, significant differences and between groups effect sizes were inconsistent between the realignment methods (Table 3). For example, the CoA_PS_ in the AP direction showed the control group had significantly greater levels of attenuation for M2 (*p* = 0.01, *d* = −0.10), M3 (*p* < 0.01, *d* = 0.62) and M4 (*p* = 0.02, *d* = 0.44) as opposed to M1 where the control group had reduced amount of attenuation and no significant difference (*p* = 0.76, *d* = −0.10).

## 4 Discussion

To our knowledge, this is the first study to test the impact of different realignment techniques upon acceleration signals obtained at different locations of the upper body during gait. Results showed that the realignment method significantly altered the values of variables derived from upper body accelerations, irrespective of group. In some cases, the amount of these alterations were such to even impact the variables ability to discriminate between PD and healthy controls.

The effect of the realignment technique on the sensitivity of upper body variables to discriminate pathology has previously been investigated, comparing realignment method M1 and M2, at sensor location (P), during treadmill walking and considering different variables than this investigation (HR being the only variable in common) [13]. Similar to this study, it was shown that tilt correction can impact discrimination amongst patient groups [13]. This agreement both confirms the need to realign raw acceleration signals and additionally shows that different realignment methods can have clear implications for interpretation of results when assessing upper body variables’ ability to detect pathological movements.

A limitation of this study was not controlling for gait speed. Although this would not impact the within group comparisons, the post hoc analysis results must be interpreted with caution as some group differences, particularly those reported for the RMS and jerk values, will merely be a reflection of gait speed [20], [22], [23]. In addition to gait speed, inconsistencies found within the literature, such as varying populations, group ages, disease severity and processing techniques do not easily allow for cross investigation comparisons. Nonetheless, the current investigation’s results did reflect similar findings to past investigations. For example, although the current investigations HR values where lower than past investigations, the PD group recorded lower HR values relative to the controls for all sensor locations and directions [5], [8].

Another limitation with the current investigation was that gold standard reference alignments were not recorded. As such, it was not possible to objectively establish which realignment method is the most accurate, or to discriminate the group differences that can be ascribed to specific aspects such as varying upper body postures, movement patterns, or solely due to signal processing choices. As a result, the recommendation for which realignment method to use for future investigations can only be prescribed based upon observations found within the current results.

The CoA, which was the only multi-segment variable investigated here, recorded the largest differences between the four methods. A possible explanation is that differences in posture at two upper body locations increased the chance of directional crosstalk impacting upon the CoA. M4 uses a uniform global reference for all locations rather than an estimated reference that may vary in accuracy due to the physical orientation of two sensors and so is theoretically better suited to use a multiple segments approach. However, M4 is limited by having to predefine and validate the global reference frame in each experimental setting prior to data collection. M2 and M3 are not limited by a predefined global reference and so are best suited across multiple settings. M3 is based on M2 but applies an additional correction of the pitch and roll angles, and has been proposed to compensate for undesired low-frequency movements during gait [15]. The amount of change observed at the head and shoulder in the investigated variables suggest that approaches similar to M3, attempting to control for erroneous movements at locations prone to cross-talk will indeed be needed when collecting data in uncontrolled environments (e.g. during free-living monitoring). Alternatively, if participants do not have significant postural differences at the location of the sensor and if erroneous movements can be controlled for by experimental design, for example instructing the participants to look forward when measuring movements of the head, or when measuring locations less prone to crosstalk such as the pelvis, M2 might still be acceptable. To support this, results showed that, with the exception of the vertical HR which did record a significant interaction but showed a consistent significant difference between the groups for all methods, no significant interaction was recorded from variables obtained at the pelvis. Therefore, inferring that, M2 can be used interchangeably from M3, and relative to the other locations investigated, the pelvis was more robust between the realignment methods.

Visual inspection of Fig. 1 shows the V acceleration signals from the pelvis were the least affected by the different realignment methods, meaning, if variables are sensitive to PD from the V signal alone, they may be able to circumvent the need to realign the signals. Previous research suggested that movements in the ML direction, or taken from the head, best highlight postural control decline, whereas pelvis movements in the V direction primarily reflect walking speed differences [20], [22], [24]. Currently it is not clear what is best between having a robust variable that requires little or no processing, or from having variables likely to be more descriptive of postural control decline as obtained using a realignment method. With additional upper body variables proposed to measure postural control in the literature (e.g. autocorrelations and Lyapunov exponent) [9], future research is needed to establish whether a particular combination of variables and realignment methods are needed to quantify PD specific impairments during gait. Nonetheless, at present, the authors predict that due to the recent abilities to use IMUs over extended periods and the interest to obtain free living gait analysis [25], the variables that are least impacted by the environment, sensor location and requires the least processing, while still being sensitive to PD, will be the most routinely utilised.

## 5 Conclusions

The current investigation is the first to show the impact of four different upper body acceleration realignment methods upon the selected upper body variables and their ability to detect PD. Realignment methods altered the results of all variables analysed, and for specific variables was able to determine their ability to highlight movements indicative of PD. Based on the strength of these results, caution is encouraged when comparing studies that use different realignment methods and that a standardisation for which method to use for each variable, sensor location and environment is required. However, prior to this, within a controlled clinical/laboratory environment and if applied to segments minimally prone to cross-talk, a tilt correction towards the vertical gravitational acceleration axis is recommended, due to its applicability to varying environments and popularity in the literature, therefore aiding a continued comparison of results.

## Conflict of interest

The authors declare that there is no conflict of interests regarding the publication of this paper.

## Figures and Tables

**Table 1:** The mean (±SD) participant characteristics and spatial-temporal gait variables for the PD and Control group.

	PD (n = 60)	Control (n = 54)	*P* (*t*-test)
Age (years)	68.5 ± 9.1	71.1 ± 6.7	0.10
Height (m)	1.68 ± 0.1	1.71 ± 0.1	0.21
Mass (kg)	77.5 ± 16.8	78.7 ± 16.5	0.69
MDS UPDRS III	26.0 ± 21.6	NA	NA
Hoehn & Yahr	HY I: 1; HY II: 52; HY III: 7	NA	NA
Number of strides	20 ± 3	21 ± 3	0.06
Step frequency (s/m)	109.6 ± 9.1	111.7 ± 9.0	0.22
Step velocity (m/s)	1.13 ± 0.23	1.29 ± 0.18	p < 0.001
Step time (s)	0.55 ± 0.05	0.54 ± 0.04	0.19
Step length (m)	0.62 ± 0.11	0.69 ± 0.08	p < 0.001
Step width (m)	0.09 ± 0.03	0.09 ± 0.02	0.39

**Table 2:** The PD and control group upper body variables values for the four realignment methods. Group by method interactions are highlighted in bold and the post hoc between group differences are indicated.

Variable			M1		M2		M3		M4	
			PD	Control	PD	Control	PD	Control	PD	Control
RMS (ms^−2^)	H	AP	0.73 ± 0.27	0.75 ± 0.22	0.67 ± 0.24	0.66 ± 0.17	0.75 ± 0.27	0.75 ± 0.22	0.68 ± 0.19	0.69 ± 0.17
		ML	0.81 ± 0.28	0.86 ± 0.22	0.81 ± 0.28	0.85 ± 0.22	0.77 ± 0.25	0.82 ± 0.20	0.68 ± 0.19	0.69 ± 0.17
		V	1.34 ± 0.49	1.68 ± 0.56	1.37 ± 0.52	1.73 ± 0.57	1.30 ± 0.48	1.64 ± 0.56	1.36 ± 0.52	1.72 ± 0.58
	S	AP	**1.02 ± 0.37**	1.29 ± 0.42**	**0.74** **±** **0.27**	**0.81** **±** **0.31**	**0.75** **±** **0.28**	**0.81** **±** **0.26**	**0.72** **±** **0.23**	**0.79** **±** **0.23**
		ML	**0.81** **±** **0.26**	**0.90** **±** **0.24**	**0.80** **±** **0.26**	**0.89** **±** **0.24**	**0.79** **±** **0.27**	**0.89** **±** 0.24*	**0.71** **±** **0.21**	**0.77** **±** **0.22**
		V	1.11 ± 0.43	1.27 ± 0.43	1.32 ± 0.49	1.63 ± 0.51	0.74 ± 0.39	0.85 ± 0.36	1.33 ± 0.49	1.65 ± 0.51
	P	AP	1.02 ± 0.39	1.24 ± 0.43	0.93 ± 0.37	1.11 ± 0.31	0.94 ± 0.39	1.21 ± 0.41	0.97 ± 0.35	1.15 ± 0.35
		ML	0.85 ± 0.32	1.01 ± 0.37	0.85 ± 0.32	1.00 ± 0.36	0.92 ± 0.35	1.07 ± 0.36	0.87 ± 0.34	1.03 ± 0.35
		V	1.41 ± 0.56	1.70 ± 0.52	1.48 ± 0.57	1.80 ± 0.59	1.38 ± 0.57	1.64 ± 0.49	1.47 ± 0.57	1.78 ± 0.58

Jerk (ms^−3^)	H	AP	19.54 ± 8.27	18.14 ± 6.17	19.37 ± 7.41	16.75 ± 6.06	20.26 ± 8.07	18.35 ± 6.67	17.91 ± 6.40	16.23 ± 5.49
		ML	**16.66** **±** **6.35**	**16.07** **±** **5.02**	**16.58** **±** **6.25**	**15.76** **±** **5.13**	**17.57** **±** **6.41**	**17.23** **±** **5.46**	**17.47** **±** **6.03**	**15.53** **±** **5.02**
		V	**39.66** **±** **14.05**	**43.65** **±** **16.56**	**39.41** **±** **15.41**	**44.42** **±** **16.10**	**38.11** **±** **13.86**	**42.49** **±** **16.34**	**39.36** **±** **15.37**	**44.35** **±** **16.29**
	S	AP	**22.97** **±** **13.16**	**25.91** **±** **10.08**	**30.67** **±** **17.49**	**31.90** **±** **16.25**	**30.84** **±** **17.70**	**31.82** **±** **15.90**	**26.63** **±** **14.28**	**28.01** **±** **12.71**
		ML	20.36 ± 9.34	21.35 ± 8.27	20.25 ± 9.37	21.10 ± 8.13	20.26 ± 9.53	21.25 ± 8.02	23.67 ± 12.05	24.56 ± 11.52
		V	39.8 ± 18.08	42.50 ± 17.47	34.16 ± 14.23	37.96 ± 13.19	26.19 ± 14.65	28.02 ± 13.14	34.74 ± 14.78	38.59 ± 13.70
	P	AP	43.40 ± 23.79	50.84 ± 25.00	39.48 ± 22.09	45.47 ± 19.11	41.15 ± 21.07	49.69 ± 23.22	39.56 ± 19.73	46.26 ± 19.85
		ML	37.49 ± 15.95	42.02 ± 17.39	37.21 ± 15.90	41.77 ± 17.02	38.78 ± 17.44	44.07 ± 17.64	37.91 ± 18.04	42.03 ± 16.61
		V	48.90 ± 23.98	56.3 ± 25.54	52.48 ± 25.24	61.19 ± 29.87	47.81 ± 24.01	54.2 ± 23.96	51.95 ± 24.35	60.1 ± 28.99

Harmonic ratio	H	AP	1.21 ± 0.35	1.38 ± 0.42	1.12 ± 0.33	1.22 ± 0.36	0.87 ± 0.38	0.89 ± 0.32	0.73 ± 0.29	0.75 ± 0.32
		ML	1.84 ± 0.57	2.02 ± 0.58	1.89 ± 0.57	2.22 ± 0.64	1.28 ± 0.64	1.24 ± 0.52	1.03 ± 0.37	1.16 ± 0.53
		V	**1.85** **±** **0.53**	**2.37** **±** 0.62**	**1.92** **±** **0.54**	**2.49** **±** 0.66**	**1.85** **±** **0.55**	**2.37** **±** 0.63**	**1.91** **±** **0.53**	**2.48** **±** 0.66**
	S	AP	**1.88** **±** **0.62**	**2.48** **±** 0.68**	**1.28** **±** **0.38**	**1.65** **±** 0.58**	**1.23** **±** **0.37**	**1.50** **±** 0.49**	**0.94** **±** **0.38**	**1.09** **±** **0.52**
		ML	1.75 ± 0.55	2.04 ± 0.50	1.85 ± 0.57	2.24 ± 0.48	1.70 ± 0.57	2.01 ± 0.57	1.05 ± 0.43	1.11 ± 0.53
		V	**1.65** **±** **0.48**	**2.17** **±** 0.68**	**2.17** **±** **0.62**	**2.91** **±** 0.77**	**1.61** **±** **0.48**	**2.14** **±** 0.68**	**2.16** **±** **0.62**	**2.92** **±** 0.78**
	P	AP	1.54 ± 0.39	2.01 ± 0.57	1.48 ± 0.39	1.95 ± 0.58	0.99 ± 0.44	1.40 ± 0.57	1.29 ± 0.44	1.56 ± 0.64
		ML	1.24 ± 0.40	1.57 ± 0.45	1.27 ± 0.40	1.60 ± 0.45	0.79 ± 0.41	1.02 ± 0.45	1.02 ± 0.39	1.12 ± 0.46
		V	**1.83** **±** **0.56**	**2.32** **±** 0.67**	**1.85** **±** **0.53**	**2.35** **±** 0.67**	**1.84** **±** **0.56**	**2.33** **±** 0.67**	**1.85** **±** **0.55**	**2.35** **±** 0.68**

Jerk ratio (dB)	H	AP/V	−3.14 ± 1.29	−3.79 ± 1.50	−3.07 ± 1.89	−4.26 ± 1.28	−2.77 ± 1.75	−3.67 ± 1.55	−3.36 ± 1.53	−4.36 ± 1.31
		ML/V	**−3.82** **±** **1.42**	**−4.27** **±** **1.25**	**−3.75** **±** **1.45**	**−4.47** **±** 1.20*	**−3.37** **±** **1.28**	**−3.86** **±** **1.38**	**−3.46** **±** **1.50**	**−4.53** **±** 1.20**
	S	AP/V	−2.46 ± 1.11	−2.14 ± 0.91	−0.66 ± 1.13	−0.94 ± 1.21	0.84 ± 1.56	0.54 ± 1.32	−1.29 ± 1.13	−1.49 ± 0.96
		ML/V	−2.94 ± 1.21	−2.97 ± 0.77	−2.36 ± 1.21	−2.60 ± 0.89	−0.89 ± 1.99	−1.07 ± 1.22	−1.8 ± 1.17	−2.10 ± 1.01
	P	AP/V	−0.62 ± 1.10	−0.53 ± 1.28	−1.36 ± 0.98	−1.24 ± 1.04	−0.69 ± 1.71	−0.47 ± 1.37	−1.24 ± 1.02	−1.12 ± 0.92
		ML/V	−1.02 ± 1.45	−1.24 ± 1.03	−1.37 ± 1.51	−1.58 ± 0.99	−0.79 ± 1.22	−0.86 ± 0.99	−1.32 ± 1.31	−1.47 ± 0.99

CoA (%)	P to H	AP	23.28 ± 27.70	35.86 ± 22.01	19.71 ± 31.37	37.86 ± 19.93	7.46 ± 43.65	32.03 ± 33.08	23.69 ± 25.71	36.45 ± 16.06
	P to S	AP	**−4.18** **±** **26.92**	**−6.82** **±** **24.26**	**15.05** **±** **23.93**	**26.32** **±** 21.90*	**10.3** **±** **38.36**	**29.69** **±** 22.23**	**21.73** **±** **20.88**	**29.5** **±** 13.29*
	S to H	AP	24.81 ± 23.74	39.46 ± 17.73	5.34 ± 26.98	13.50 ± 21.55	−5.47 ± 32.89	3.75 ± 23.78	2.41 ± 18.85	9.90 ± 15.28
	P to H	ML	−3.71 ± 38.15	7.41 ± 29.08	−4.08 ± 38.43	7.61 ± 29.55	9.29 ± 32.46	17.41 ± 23.71	14.11 ± 29.26	27.37 ± 23.09
	P to S	ML	−0.66 ± 30.28	4.90 ± 24.39	−0.80 ± 30.66	5.29 ± 24.57	8.54 ± 28.77	11.59 ± 21.79	12.88 ± 22.59	21.70 ± 19.04
	S to H	ML	−1.75 ± 16.89	3.14 ± 13.68	−2.00 ± 16.92	2.98 ± 13.86	0.35 ± 22.27	6.30 ± 15.89	2.45 ± 16.79	7.63 ± 16.89
	P to H	V	2.95 ± 13.04	1.12 ± 9.96	6.84 ± 11.75	3.63± 7.86	3.09 ± 18.56	−0.09 ± 13.52	6.44 ± 12.42	3.67 ± 8.14
	P to S	V	**18.99** **±** **19.34**	**25.25** **±** 11.78*	**9.71 ± 9.74**	**8.9 ± 7.58**	**45.06** **±** **21.87**	**48.15** **±** **14.54**	**8.66** **±** **10.68**	**7.33 ± 6.13**
	S to H	V	−24.12 ± 27.33	−35.36 ± 24.45	−3.2 ± 7.15	−6.35 ± 11.77	−81.74 ± 57.2	−90.44 ± 53.51	−2.48 ± 7.56	−4.01 ± 6.47

a. **bold** indicates the variables where a significant interaction effect was recorded.

b. *highlights a significant difference between groups at the *p* < 0.05 level.

c. ** highlights a significant difference between groups at the *p* < 0.001 level.

d. *H* *=* *head level. S* *=* *shoulder level. P* *=* *pelvis level.*

e. *AP* *=* *anterior-posterior. ML* *=* *medio-lateral. V* *=* *vertical.*

**Table 3:** The post-hoc paired sample *t*-test *p* values and effect size (*d*) values for all variables and realignment methods where an interaction occurred.

Variable			M1		M2		M3		M4	
RMS			*p*	*d*	*p*	*d*	*p*	*d*	p	*d*
	S	AP	<0.001	0.68	0.246	0.68	0.323	0.19	0.103	0.31
	S	ML	0.058	0.36	0.070	0.36	0.032	0.41	0.182	0.25

J	H	ML	0.590	−0.10	0.452	−0.10	0.764	−0.06	0.069	−0.35
	H	V	0.170	0.26	0.095	0.26	0.127	0.29	0.098	0.32
	S	AP	0.191	0.25	0.701	0.25	0.760	0.06	0.590	0.10

HR	H	V	<0.001	0.89	<0.001	0.89	<0.001	0.89	<0.001	0.94
	S	AP	<0.001	0.93	<0.001	0.93	<0.001	0.61	0.093	0.32
	S	V	<0.001	0.89	<0.001	0.89	<0.001	0.89	<0.001	1.09
	P	V	<0.001	0.79	<0.001	0.79	<0.001	0.80	<0.001	0.81

JR	ML/V	ML	0.077	−0.34	0.006	−0.34	0.053	−0.37	<0.001	−0.79

CoA	P to S	AP	0.589	−0.10	0.011	−0.10	<0.001	0.62	0.022	0.44
	P to S	V	0.043	0.39	0.628	−0.09	0.386	0.17	0.430	−0.15

*a.* H = head level. S = shoulder level. P = pelvis level.

*b.* AP = anterior-posterior. ML = medio-lateral. V = vertical.
